# The role of self-reported stressors in recovery from Exhaustion Disorder: a longitudinal study

**DOI:** 10.1186/s12888-022-04172-y

**Published:** 2022-08-05

**Authors:** Britta Eklöf, Hanna Larsson, Susanne Ellbin, Ingibjörg H. Jonsdottir, Siobhan O’Dwyer, Caroline Hansson

**Affiliations:** 1 Institute of Stress Medicine, Region Västra Götaland, Carl Skottsbergs gata 22B, SE-413 19 Gothenburg, Sweden; 2 School of Public Health and Community Medicine, Institute of Medicine, Sahlgrenska Academy, Gothenburg University, Gothenburg, Sweden; 3 College of Medicine and Health, University of Exeter Medical School, University of Exeter, Exeter, UK; 4 Department of Psychiatry and Neurochemistry, Institute of Neuroscience and Physiology, Sahlgrenska Academy, Gothenburg University, Gothenburg, Sweden

**Keywords:** Exhaustion, Burnout, Stressors, Adverse childhood experiences, Mixed methods, Carers, Caregivers, Parent Carers

## Abstract

**Background:**

Exhaustion disorder (ED) is a stress-induced disorder characterized by physical and mental symptoms of exhaustion that can be long-lasting. Although stress exposure is essential for the development of ED, little is known regarding the role of stressors in the maintenance of ED. The aim of the study was to investigate the role of work-related stressors, private-related stressors, and adverse childhood experiences in long-term recovery from ED.

**Methods:**

A mixed methods design was used. The design was sequential, and data analysis was performed in two parts, where the first part consisted of qualitative analysis of patient records, and the second part consisted of statistical analysis of the data retrieved from the qualitative coding. Patient records from 150 patients with ED was analysed regarding work-related stressors, private-related stressors, and adverse childhood experiences. For each patient, two patient records were analysed, one from the time of diagnosis (baseline) and one from the follow-up clinical assessment, 7–12 years after diagnosis (follow-up). Out of the 150 patients, 51 individuals still fulfilled the diagnostic criteria for ED at follow-up (ED group) and 99 individuals no longer fulfilled the diagnostic criteria and were thus considered recovered (EDrec). Percentages in each group (ED and EDrec) reporting each stressor at baseline and follow-up were calculated as well as the differences in percentage points between the groups along with the 95% confidence intervals for the differences.

**Results:**

At baseline, significantly more EDrec patients reported quantitative demands (73% EDrec, 53% ED) and managerial responsibilities (14% EDrec, 2% ED). Private-related stressors did not differ at baseline. At follow-up, significantly more ED patients reported managerial responsibilities (8 ED, 0% EDrec) and caregiver stress (child) (24% ED, 6% EDrec) and significantly more EDrec patients reported caregiver stress (parent) (6% EDrec, 0% ED). There were no differences regarding adverse childhood experiences.

**Conclusions:**

The main conclusion is that neither adverse childhood experiences nor any of the stressors at baseline are associated with long-term ED. Ongoing stressors related to having responsibility for other people, such as managerial responsibilities or caring for a child with a chronic disease or psychiatric disorder, may be associated with long-term exhaustion.

## Background

Stress-related mental health problems are a rising problem in Europe and takes a significant social and economic toll, via lost productivity and increased strain on health and social care systems [1–3]. In Sweden, stress-related diagnoses are the fastest growing causes of sick leave and account for approximately half of all cases of sick leave attributed to psychiatric diagnoses [4].

A variety of partly overlapping concepts are being used in the literature on stress-related mental health problems. Exhaustion disorder (ED) is a criteria-based diagnosis published by the National Board of Health and Welfare in Sweden in 2003 [5] and is used for severe cases of exhaustion caused by stressors present for at least six months (for an overview of the development of the diagnosis, see [6, 7]). The ED diagnosis overlaps with the burnout concept and in some cases the term clinical burnout is used. Burnout, however, is a psychological construct based solely on work-related stress and cannot be used as a diagnostic tool in clinical practice [8]. Core symptoms of ED include extreme fatigue, cognitive impairments, sleep disturbances, less resistance to stress, and somatic symptoms such as stomach problems and chronic pain (Table 1).

**Table 1:** Diagnostic criteria for Exhaustion Disorder according to the National Board of Health and Welfare (2003)

A. Physical and mental symptoms of exhaustion with minimum two weeks duration. The symptoms have developed in response to one or more identifiable stressors which have been present for at least 6 months
B. Markedly reduced mental energy, which is manifested by reduced initiative, lack of endurance, or increase of time needed for recovery after mental efforts
C. At least four of the following symptoms have been present most of the day, nearly every day, during the same 2-week period: 1. Persistent complaints of impaired memory 2. Markedly reduced capacity to tolerate demands or to work under time pressure 3. Emotional instability or irritability 4. Insomnia or hypersomnia 5. Persistent complaints of physical weakness or fatigue 6. Physical symptoms such as muscular pain, chest pain, palpitations, gastrointestinal problems, vertigo, or increased sensitivity to sounds
D. The symptoms cause clinically significant distress or impairment in social, occupational, or other important areas of functioning
E. The symptoms are not due to the direct physiological effects of a substance (e.g. a drug of abuse, a medication) or a general medical condition (e.g. hypothyroidism, diabetes, infectious disease)
F. If criteria for major depressive disorder, dysthymic disorder or generalized anxiety disorder are met, exhaustion disorder is set a comorbid condition

Recovery from stress-related exhaustion can be a lengthy process and many patients experience long-lasting functional impairment related to cognition and/or fatigue several years after seeking care [6, 9]. A recent longitudinal study showed that one-third of patients still met the diagnostic criteria seven years after being diagnosed with and treated for ED, and the majority still struggled with ongoing symptoms such as decreased stress tolerance, fatigue and cognitive problems [6]. In order for clinicians to identify individuals at risk of long-lasting exhaustion and to prevent an extended rehabilitation process, there is an urgent need to investigate which factors contribute to delayed recovery.

For a diagnosis of ED the exhaustion must have developed because of identifiable stressor(s) present for at least six months [5]. Prior research has shown that both work-related exposure (such as high job demands and low job control) and personal life stressors (such as stressful life events, high caregiving burden, and family demands) increase the risk of developing stress-related disorders, burnout and/or other negative health outcomes [10–12]. This is confirmed by our previous study showing that patients with ED report exposure to stressors in both their work (work-related) and private lives (private-related) as contributing to the development of their exhaustion [13]. Moreover, severe stressors during childhood (such as physical, emotional or sexual abuse, or growing up with a family member with mental illness or substance abuse), referred to as adverse childhood experiences [14], have been shown to be associated with increased vulnerability for stress-related disorders [14], depressive symptoms [15], and psychological distress [16]. Although stress exposure is obviously central for the development of ED, little is known regarding the role of stressors in the process of recovering from stress-related exhaustion.

The present study explores the role of work-related stressors, private-related stressors, and adverse childhood experiences in long-term recovery from ED. More specifically, the aim was to determine whether, at the time of diagnosis and/or at the seven years follow-up, stressors differ between patients who develop long-term exhaustion (ED) and patients who recover (EDrec). The secondary aim was to determine if adverse childhood experiences differ between patients who develop long-term exhaustion (ED) and patients who recover (EDrec).

## Method

### Participants and setting

This study is part of a longitudinal study of ED conducted at the Institute of Stress Medicine, a specialist outpatient clinic for patients with ED in Gothenburg, Sweden. Patients were referred to the clinic by primary health care units or occupational health service centres. The initial criteria for receiving treatment at the clinic were that the patient fulfilled the diagnostic criteria for ED (Table 1), was of working age (18–64 years old) and had not been on sick-leave for more than 6 months prior to referral to the clinic. Differential diagnostic procedures were conducted during the first visit to the clinic excluding patients with generalized pain, fibromyalgia, chronic fatigue syndrome/myalgic encephalomyelitis, thyroid disease, vitamin B_12_ deficiency, obesity, alcohol/drug addiction, psychiatric illness other than depression and anxiety, and other somatic diseases that could explain fatigue. All patients included in this follow-up initially received treatment at the clinic for a period of approximately 18 months. The treatment has been described in detail previously [17].

**Fig. 1:**
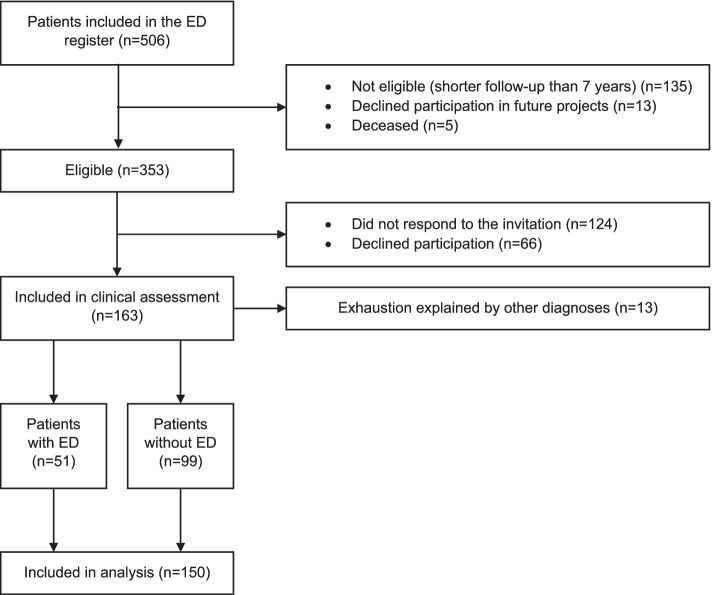
Flow-chart of included participants

All patients previously treated at the clinic were invited to be included in a follow-up register. Furthermore, all patients for which seven years or more had passed since their first visit to the clinic (*n* = 353) were invited to participate in a follow-up clinical assessment to assess residual stress-related exhaustion (Fig. 1). The seven years limit was chosen based on clinical experience, showing that many ED patients have symptoms lasting several years after completion of treatment. Around half or 163 patients (46%) agreed to participate in the clinical assessments. The patients that agreed to participate (included in clinical assessment, *n* = 163) were significantly older at baseline (mean age 44 years, SD 9.6) than the patients that were eligible, but did not agree to participate or did not answer the invitation (drop-out group, *n* = 190) (mean age 41 years, SD 9.0, *p* = 0.003). There were also significantly more women in the participating group (77%) than in the drop-out group (67%, *p* = 0.041). The groups did not differ at baseline regarding self-reported symptoms of burnout, anxiety, or depression (data not shown). Out of the 163 patients that agreed to participate in the clinical assessment, 51 individuals were judged to still fulfil the diagnostic criteria for ED (ED group) and 99 individuals were judged to no longer fulfil the diagnostic criteria for ED and were thus considered recovered (EDrec group). Thirteen individuals were judged to suffer from exhaustion due to other somatic or psychiatric diagnoses that excluded them from fulfilling the diagnostic criteria for ED [6], and so were excluded from the present study. Thus, a total of 150 patients were included in the present study. The ED and EDrec groups did not differ at baseline on sex, age, marital status and education level (Table 2).

**Table 2:** Baseline characteristics of the patients included in the present study

	**Total (*n* = 150)**	**ED (*n* = 51)**	**EDrec (*n* = 99)**	***P*-value**
Sex				.927
- Women % (n)	78% (117)	78% (40)	78% (77)	
- Men % (n)	22% (33)	22% (11)	22% (22)	
Age mean (SD)	44 (9.6)	43 (9.4)	44 (9.8)	.578
Marital status				.142
- Married/co-living % (n)	74% (111)	67% (34)	78% (77)	
- Dating/single/other % (n)	26% (39)	33% (17)	22% (22)	
Education				.318
- Higher % (n)	71% (107)	77% (39)	69% (68)	
- Lower % (n)	29% (43)	24% (12)	31% (31)	

Of total 150 patients, 51 individuals still fulfilled the diagnostic criteria for Exhaustion Disorder at the follow-up clinical assessment, 7–12 years later (ED), and 99 individuals no longer fulfilled the diagnostic criteria (EDrec)

Independent samples t-test was used to analyse differences regarding mean age between the groups and chi-square tests were used for comparing the distribution of sex, marital status, and educational level

Higher education is defined as ≥ 1 year of college education

### Measurements

#### Demographic information

Information regarding sex, age, marital status, and education level was collected during the patients’ first visit to the clinic.

#### Work-related and private-related stress exposure and adverse childhood experiences

Patient records from the patient’s first visit to the clinic (baseline) and their follow-up clinical assessment 7–12 years later (*M* = 9.36, *SD* = 1.61) were used to extract information regarding stress exposure and adverse childhood experiences. Both visits lasted approximately 90 min and were conducted by senior physicians at the clinic. During these visits, physicians collected each patient’s current and past medical and social history. Exposure to stressors was examined through a procedure where the physician exemplified different types of stressors (physical/environmental, emotional, and social, both work-related and private-related) and then asked the patient about the stressors he/she had experienced. The physicians wrote a detailed summary in the patient’s records immediately after each visit. Baseline stressors were coded from the patient’s first visit to the clinic and follow-up stressors were coded from their follow-up clinical assessment. Adverse childhood experiences were coded based on information from both visits.

### Procedure and analysis

A mixed methods design [18] was applied in the present study. The design was sequential, and data analysis was performed in two parts. The first part consisted of qualitative analysis of the patient records from the clinical assessments at baseline and follow-up, and the second part consisted of statistical analysis of the data retrieved from the qualitative coding. The qualitative analysis was performed in accordance with content analysis [19], using a combination of inductive and deductive coding. Twenty randomly selected patient records were coded independently by the authors BE, HL, and SE, using an inductive approach. All meaning units [20] describing stressors or adverse childhood experiences reported by the participants were highlighted and given codes. The size of the meaning units varied between a few words to multiple full sentences. Only manifest content was coded. The codes were then compared between coders and grouped into higher order categories of stressors and adverse childhood experiences, resulting in a preliminary coding scheme. Stressors were defined as events or circumstances reported by the patient as causing significant stress. Only stressors reported as ongoing or recent at the time of the visit and having contributed negatively to the patient’s health were included. Each stressor was judged as either present or not, and thus not quantified even if mentioned several times. For instance, having multiple relational problems was coded the same way as having one relational problem. Similarly, if stressors were interconnected, such that one stressor led to another (e.g. deficient leadership led to a lack of organisational structure and conflict), each stressor reported by the participant was coded separately. Adverse childhood experiences were defined as events or circumstances during the patient’s childhood which were described by the patient as causing significant harm, or that were judged, by the coders, as potentially harmful based on the severity of the event. The categories reflected the codes generated from the inductive coding process, but prior understanding of categories identified by previous research [13] guided the conceptualization and naming of the categories. Examples of included and excluded codes for each category were noted. An additional eleven patient records were coded, resulting in minor adjustments to the coding scheme. After coding these eleven records, the coders judged the material to be saturated and a final coding scheme was decided upon. All 150 patient records were then coded using the final coding scheme. The coding scheme was used to establish a dichotomous variable, where the presence (= 1) or not presence (= 0) of each stressor or adverse childhood experience was noted for each patient at each timepoint. This dichotomous variable was then used for the quantitative analysis.

### Statistical analysis

Descriptive statistics are presented as frequencies and percentages for categorical and dichotomous variables and as mean and standard deviation (SD) for continuous variables. Independent samples t-test was used to analyse differences regarding mean age between the groups and chi-square tests were used for comparing the distribution of sex, marital status, and educational level. Significance levels were set to *p* < 0.05. For each stressor, percentages in each group (ED and EDrec) reporting the stressor were calculated as well as the differences in percentage points between the groups at each time point (baseline and follow-up) along with the 95% confidence intervals (CI) for the differences. All analyses were carried out using SPSS version 25.

### Ethics approval and consent to participate

All participants gave their written informed consent before entering the study. This study was performed in accordance with Declaration of Helsinki. The protocol including all ethical aspects according to the Declaration of Helsinki was approved by the Regional ethical review board in Gothenburg Sweden, which is a part of the Swedish national committee for ethical approval, 2015–10-16 (Dnr 668–15).

## Results

### Work-related stress at baseline

The work-related stressors identified in the qualitative analysis resulted in a total of 12 categories (see Table 3 for list and definitions). There were two significant differences between ED and EDrec patients at baseline (see Table 4). Significantly more EDrec patients reported quantitative demands (73% compared with 53% ED) and managerial responsibilities (14% compared with 2% ED). For both groups, the most frequently reported stressors at baseline were quantitative demands (53% ED; 73% EDrec), conflicts/bullying (25% ED; 29% EDrec), changing or lack of organizational structure (24% ED; 24% EDrec), emotional demands (24% ED; 19% EDrec) and deficient leadership (18% ED; 29% EDrec).

**Table 3:** Description of the categories of work-related stressors

Category	Description
Quantitative demands	Issues related to the amount, pace, and/or complexity of work, such as having a high workload, working under time pressure, performing cognitively demanding tasks, or working at multiple workplaces
Conflicts or bullying	Conflicts with colleagues, subordinates, or managers, feeling excluded at work, being subject to bullying or harassment, or working in a conflict-filled environment
Changing or lack of organizational structure	Lack of structure at work due to re-organizations or other reasons, having a high turnover of employees at one’s workplace, having unclear tasks or role at work
Deficient leadership	Issues related to leadership at work, including deficient or absent leadership, having a high turnover of managers, or lacking trust in one’s manager
Emotional demands	Work described as emotionally demanding or exhaustive due to for example care-giving tasks, dealing with complaints from clients, ethical stress, or not having enough competence for one’s assignments
Irregular working hours	Irregular or inconvenient working hours, long-distance commuting, travelling for work, or working overtime
Managerial responsibilities	Issues related to having managerial responsibilities at work
Job insecurity	Being dismissed from work or worrying about losing one’s job, issues related to having insecure or short-term employments
Discontent at work	General discontent or aversion towards the workplace, being preoccupied by thoughts about quitting or changing jobs, reporting one’s work as dull or monotonous
Deficiencies in work environment	Issues related to the physical or digital work environment, such as repeatedly having to change workspace, not having the necessary tools to perform one’s job, or having extensive problems with IT
Lack of reward	Perceived lack of reward at work, such as getting promises about a raise or other benefits that are not followed through, not getting the same raise or benefits as colleagues, feelings of working hard without getting anything back
Lack of autonomy or control	Having a controlling or rigid employer, not being allowed to participate in important changes regarding one’s work, being given tasks against one’s will

**Table 4:** Work-related stressors reported by the ED and EDrec groups at baseline and at follow-up

Work-related stressor	Time	ED % (n)	EDrec % (n)	Difference in %-points (95% CI)
Quantitative demands	T1	53% (27)	73% (72)	-19.8 (-36.4; -3.2)
	T2	16% (8)	16% (16)	-0.5 (-13.0; 12.1)
Conflicts or bullying	T1	25% (13)	29% (29)	-3.8 (-19.2; 11.6)
	T2	8% (4)	4% (4)	3.8 (-3.9; 11.5)
Changing or lack of organizational structure	T1	24% (12)	24% (24)	-0.7 (-15.4; 13.9)
	T2	8% (4)	7% (7)	0.8 (-8.2; 9.7)
Deficient leadership	T1	18% (9)	29% (29)	-11.6 (-25.7; 2.4)
	T2	8% (4)	3% (3)	4.8 (-3.5; 13.1)
Emotional demands	T1	24% (12)	19% (19)	4.3 (-9.5; 18.2)
	T2	10% (5)	3% (3)	6.8 (-2.3; 15.9)
Irregular working hours	T1	8% (4)	15% (15)	-7.3 (-17.7; 3.1)
	T2	2% (1)	2% (2)	0.0 (-4.9; 4.7)
Managerial responsibilities	T1	2% (1)	14% (14)	-12.2 (-20.1; -4.2)
	T2	8% (4)	0% (0)	7.8 (0.2; 15.5)
Job insecurity	T1	12% (6)	5% (5)	6.7 (-3.4; 16.8)
	T2	4% (2)	3% (3)	0.9 (-5.3; 7.0)
Discontent at work	T1	6% (3)	10% (10)	-4.2 (-13.8; 5.4)
	T2	0% (0)	2% (2)	-2.0 (-4.8; 0.8)
Deficiencies in work environment	T1	8% (4)	2% (2)	5.8 (-2.3; 13.9)
	T2	4% (2)	2% (2)	1.9 (-3.6; 7.4)
Lack of reward	T1	4% (2)	6% (6)	-2.1 (-9.8; 5.6)
	T2	2% (1)	1% (1)	1.0 (-3.0; 4.9)
Lack of autonomy or control	T1	2% (1)	3% (3)	-1.1 (-6.6; 4.5)
	T2	0% (0)	0% (0)	-

ED = participants that still fulfil the diagnostic criteria for Exhaustion Disorder at follow-up, i.e. the Exhaustion Disorder group (*n* = 51)

EDrec = participants that no longer fulfil the diagnostic criteria for Exhaustion Disorder at follow-up, i.e. the recovered group (*n* = 99)

T1 = Timepoint 1, baseline, i.e. at the time of diagnosis

T2 = Timepoint 2, follow-up, i.e. 7–12 years after diagnosis

Percentages in each group (ED and EDrec) reporting each stressor were calculated as well as the differences in percentage points between the groups at each time point (baseline and follow-up) along with the 95% confidence intervals (CI) for the differences

### Work-related stress at follow-up

There was one significant difference between ED and EDrec patients at follow-up (see Table 4). Significantly more ED patients reported managerial responsibilities (8% compared with 0% EDrec). The most frequently reported stressor at follow-up was quantitative demands (16% in both groups).

### Private-related stress at baseline

The private-related stressors identified in the qualitative analysis resulted in a total of 15 categories (see Table 5 for list and definitions). There were no significant differences between ED and EDrec patients at baseline regarding the percentage of patients reporting different private-related stressors (see Table 6). The most frequently reported stressors in both groups at baseline were relational problems (29% ED; 29% EDrec), high inner demands (31% ED; 26% EDrec) and worries about family member (22% ED; 25% EDrec).

**Table 5:** Description of the categories of private-related stressors

Category	Description
Relational problems	Relational conflicts, stressful separation or divorce, discontent with one’s role in the relationship, stress concerning long-distance relationships
Worries about family member	Extensive worries about family member with social problems such as addiction, psychiatric disorders, or somatic conditions such chronic as fatigue syndrome, diabetes, or cancer
High inner demands	Having high demands regarding performance at work or in private life, struggling with setting boundaries, taking on more tasks than what is needed or possible, being self-critical or sensitive to critique from others, over-compensating for personal shortcomings
Personal health issues	Issues concerning personal health described as increasing the perceived stress load, for example by being restricted in daily life by chronic pain or fatigue, going through extensive rehabilitation after an injury, or worrying excessively about one’s health
Caregiver stress (child)	Being the primary caregiver for a child with psychiatric disorders, such as ADHD, or chronic illness, such as narcolepsy
Financial worries	Suffering from financial strain or extensive worries regarding one’s financial situation
Residential worries	Issues related to one’s housing situation, for example having to perform extensive maintenance on one’s home, worrying about future renovations, working close to home and being reminded of work at home
Caregiver stress (parent)	Caring extensively for one’s parents or other close relative with psychiatric disorders or chronic somatic disorders for a longer period
Death of a family member	Loss of family member or close friend described as causing significant stress
Caregiver stress (partner)	Caring extensively for one’s partner with psychiatric disorders or chronic somatic disorders for a longer period
Change in family composition	Issues related to having a baby, adult children moving out of the household, or moving in with a partner and their children
Single parent	Having sole responsibility for household work and childcare, either by being a single parent or as a result of not getting support from one’s partner
Abuse/harassment	Being the victim of psychological, physical, or sexual abuse
Stressful contact with authorities	Issues related to contact with the Social Insurance Agency, health care providers, or other authorities

**Table 6:** Private-related stressors reported by the ED and EDrec groups at baseline and at follow-up

Private-related stressor	Time	ED % (n)	EDrec % (n)	Difference in %-points (95% CI)
Relational problems including separation/divorce	T1	29% (15)	29% (29)	0.1 (-15.5; 15.7)
	T2	27% (14)	20% (20)	7.2 (-7.1; 21.6)
Worries about family member	T1	22% (11)	25% (25)	-3.7 (-18.3; 10.9)
	T2	18% (9)	20% (20)	-2.6 (-16.1; 11.0)
High inner demands	T1	31% (16)	26% (26)	5.1 (-10.3; 20.5)
	T2	12% (6)	12% (12)	-0.4 (-11.5; 10.8)
Personal health issues	T1	8% (4)	12% (12)	-4.3 (-14.8; 6.3)
	T2	18% (9)	10% (10)	7.5 (-4.8; 19.9)
Caregiver stress (child)	T1	16% (8)	8% (8)	7.6 (-4.0; 19.2)
	T2	24% (12)	6% (6)	17.5 (4.6; 30.4)
Financial worries	T1	10% (5)	9% (9)	0.7 (-9.3; 10.7)
	T2	10% (5)	6% (6)	3.7 (-5.2; 12.7)
Residential worries	T1	8% (4)	4% (4)	3.8 (-3.9; 11.5)
	T2	10% (5)	4% (4)	5.8 (-3.5; 15.0)
Caregiver stress (parent)	T1	2% (1)	5% (5)	-3.1 (-9.8; 3.6)
	T2	0% (0)	6% (6)	-6.1 (-10.8; -1.3)
Death of a family member	T1	10% (5)	2% (2)	7.8 (-1.1; 16.7)
	T2	4% (2)	1% (1)	2.9 (-2.9; 8.8)
Caregiver stress (partner)	T1	6% (3)	3% (3)	2.9 (-3.9; 9.6)
	T2	4% (2)	1% (1)	2.9 (-2.9; 8.8)
Change in family composition	T1	8% (4)	4% (4)	3.8 (-3.9; 11.5)
	T2	0% (0)	1% (1)	-1.0 (-3.8; 1.8)
Single parent	T1	4% (2)	5% (5)	-1.1 (-8.4; 6.1)
	T2	2% (1)	1% (1)	1.0 (-3.0; 4.9)
Abuse/harassment	T1	6% (3)	5% (5)	0.8 (-6.9; 8.5)
	T2	0% (0)	0% (0)	-
Stressful contact with authorities	T1	2% (1)	2% (2)	-0.1 (-4.9; 4.7)
	T2	8% (4)	1% (1)	6.8 (-1.0; 14.7)

ED = participants that still fulfil the diagnostic criteria for Exhaustion Disorder at follow-up, i.e. the Exhaustion Disorder group (*n* = 51)

EDrec = participants that no longer fulfil the diagnostic criteria for Exhaustion Disorder at follow-up, i.e. the recovered group (*n* = 99)

T1 = Timepoint 1, baseline, i.e. at the time of diagnosis

T2 = Timepoint 2, follow-up, i.e. 7–12 years after diagnosis

Percentages in each group (ED and EDrec) reporting each stressor were calculated as well as the differences in percentage points between the groups at each time point (baseline and follow-up) along with the 95% confidence intervals (CI) for the differences

### Private-related stress at follow-up

There were two significant differences between ED and EDrec patients at follow-up (see Table 6). Significantly more ED patients reported caregiver stress (child) (24% compared with 6% EDrec) and significantly more EDrec patients reported caregiver stress (parent) (6% compared with 0% ED). The most frequently reported stressors in the ED group at follow-up were relational problems (27%), caregiver stress (child) (24%), worries about family member (18%) and personal health issues (18%). The most frequently reported stressors in the EDrec group at follow-up were relational problems (20%), worries about family member (20%) and higher inner demands (12%).

### Adverse childhood experiences

The adverse childhood experiences identified in the qualitative analysis resulted in a total of nine categories (see Table 7 for list and definitions). There were no significant differences between ED and EDrec patients regarding the percentage of patients reporting different adverse childhood experiences (see Table 8). The most common adverse childhood experience in both groups was social/psychological problems in family, which was reported by almost one-quarter of all patients (22% ED; 26% EDrec).

**Table 7:** Description of the categories of adverse childhood experiences

Category	Description
Social/psychological problems in family	Psychiatric disorders, addiction, or severe financial issues among members of the household. Witnessing violence between family members
Separation/conflicts between parents	Intense conflicts or separation/divorce between parents
Bullying	Being bullied by peers
School problems	Problems managing school, having to retake a year or not graduating
Physical abuse	Being physically abused by parents or other family members
Early separation from parent	Being separated from parents due to foster care placement, being placed in the care of a relative, the parent dying, or other reasons
Psychological abuse	Being verbally abused by parents/other family members or reporting distress due to their unpredictable and/or explosive temper
Emotional neglect	Not getting emotional needs met during childhood, for example describing parents as cold, not getting affection, or having to be self-reliant at an early age
Sexual abuse	Being sexually abused or subject to inappropriate sexual conduct

**Table 8:** Adverse childhood experiences reported by the ED and EDrec groups

Adverse childhood experience	ED % (n)	EDrec % (n)	Difference in %-points (95% CI)
Social/psychological problems in family	22% (11)	26% (26)	4.7 (-10.1; 1.9)
Separation/conflicts between parents	10% (5)	10% (10)	0.3 (-10.0; 10.6)
Bullying	8% (4)	9% (9)	1.2 (-8.4; 10.9)
School problems	8% (4)	9% (9)	1.2 (-8.4; 10.9)
Physical abuse	8% (4)	8% (8)	0.2 (-9.1; 9.5)
Early separation from parent	10% (5)	6% (6)	-3.7 (-12.7; 5.2)
Psychological abuse	8% (4)	4% (4)	-3.8 (-11.5; 3.9)
Emotional neglect	6% (3)	4% (4)	-1.8 (-9.1; 5.4)
Sexual abuse	2% (1)	3% (3)	1.1 (-4.4; 6.6)

ED = participants that still fulfil the diagnostic criteria for Exhaustion Disorder at follow-up, i.e. the Exhaustion Disorder group (*n* = 51)

EDrec = participants that no longer fulfil the diagnostic criteria for Exhaustion Disorder at follow-up, i.e. the recovered group (*n* = 99)

For each adverse childhood experience, percentages in each group (ED and EDrec) were calculated as well as the differences in percentage points between the groups along with the 95% confidence intervals (CI) for the differences

## Discussion

### Main findings

At the time of diagnosis, there was no difference in the frequency with which private-related stressors were reported by patients who would go on to recover from ED and patients who would not. For workplace stressors, quantitative demands and managerial responsibilities were reported more frequently at the time of diagnosis (baseline) by those who would recover than those who would not. At long-term follow-up, caregiver stress (child) and managerial responsibilities were reported more frequently by those who had not recovered, but caregiver stress (parent) was reported more frequently by those who had recovered. There were no differences between the groups regarding adverse childhood experiences.

### Work-related stress

The most frequently reported stressor in both groups at baseline was high quantitative demands at work. This is in line with previous research identifying quantitative demands at work as the most frequently reported stressor responsible for the development of ED [13]. Moreover, a systematic review and meta-analysis found that high workload increases the risk of developing exhaustion [11]. These data suggest that quantitative demands at work is an important driving force regarding development of ED. However, this stressor was significantly more common in the EDrec group than in the ED group, indicating that high quantitative demands at baseline is not a risk factor for long-term exhaustion. At follow-up, there was no longer any differences between the groups and quantitative demands at work was reported by only 16% in both groups.

While managerial responsibilities were significantly more common in the EDrec group at baseline, this stressor was significantly more common in the ED group at follow-up. Managerial responsibilities have previously been reported to be an important stressor for the onset of ED [13] and our data suggest that this stressor, if added to a person that has already developed ED, is associated with long-term exhaustion. One of the main theoretical models for explaining work-related psychological distress is the job demand-control model, which hypothesises that high decision authority moderates high demands at work. However, this model has been questioned in empirical studies. Interestingly, a large longitudinal study showed that higher decision authority increases the risk of psychological distress [21]. This is contrary to the job demand-control model, but in line with our results, as managerial responsibilities likely involve high decision authority. However, not all managers experience high decision authority. Studies have shown that first-line human service managers generally have high job demands and restricted decision authority [22]. The fact that ongoing stress in the form of managerial responsibilities at follow-up was associated with higher risk of long-term exhaustion suggests that some managers included in this study seem to be dealing with a strained work situation without enough resources. Therefore, it is important to improve the work environment for all employees, including managers.

### Private-related stress

Private-related stress exposure did not differ between the groups at the time of diagnosis, indicating that this type of stress exposure does not explain the long-term exhaustion seen in the non-recovered group. Likewise, most private-related stressors were similar between the groups at the seven years follow-up. One important exception is caregiver stress. While stress associated with caring for a parent was more common in the recovered group, a significantly larger proportion of the non-recovered group reported stress associated with caring for a child at follow-up. In fact, 1 in 4 patients who were still exhausted at the seven years follow-up reported caring for a child with a psychiatric disorder or chronic illness to be a stressor of relevance for their situation. Caring for a parent who is elderly or has a condition such as dementia, is usually a time-limited experience and options for reprieve (namely long-term residential aged care) are readily available and socially sanctioned in the global north [23, 24]. Caring for a child with a long-term illness or disability however, is a life-long commitment, the stress of which often increases as the child and the parent age (particularly when the child transitions out of education and children’s services, or the parent is faced with their own mortality) and for which there are few, if any, options for permanent reprieve [25–29]. Identifying parents of children with long-term illnesses and disabilities at the time of the ED diagnosis may be an important first step towards preventing the development of long-term exhaustion in this group, by creating opportunities to provide additional support.

### Adverse childhood experiences

There were no significant differences between the groups regarding adverse childhood experiences. Based on these results, exposure to adverse childhood experiences does not seem to be associated with the persistence of ED. There are no previous studies examining the association between ED and adverse childhood experiences, and it is therefore not known whether adverse childhood experiences have any impact on the development of ED. Previous studies have shown that the association between childhood adversities and psychiatric disorders are much stronger in relation to the *onset* of the disorder, rather than to its *persistence* [30, 31]. Possibly, this could be the case for ED as well and needs to be further studied.

### Methodological considerations

A major strength with this study is its longitudinal design, since there are few studies on patients with clinical burnout or ED with such a long-term follow-up. Moreover, the mixed-method design allowed for using data from personalized interviews, and still include a large study group of 150 patients. However, several methodological considerations need to be discussed. One limitation with using patient records is that they are not first-hand data, but represent the physicians’ understanding of their patients’ narratives. There is therefore a risk of bias in what the physicians included in the records. There is also a potential bias in reporting of private-related stress at follow-up due to it being easier for patients to report private related stress at follow-up when they had already met, and potentially felt more comfortable around, the physician. Another limitation is that the dichotomous assessment that we created to enable quantitative analyses does not capture the full complexity of experiences such as adverse childhood experiences, work- or private-related stressors. This dichotomous assessment only captures the type of stressor or adverse childhood experience, but not the frequency or severity. However, all such biases can be assumed to be distributed equally between the two groups. The physicians did not follow a structured protocol when asking about adverse childhood experiences, which means that the emphasis placed on this part of the clinical assessment might have differed between physicians and potentially affected the patients’ willingness to report adverse childhood experiences. Further, this part of the data is based on retrospective reporting, which also entails a risk of recall bias. Using a structured protocol would have standardized the data collection, but also comes with the risk of limiting the scope of data being collected. The fact that the patients were asked twice about adverse childhood experiences, both at baseline and at follow-up, mitigates this concern to some extent. Stressors were identified following a semi-structured procedure where the physician exemplified different types of stressors and then asked the patient about the stressors that he/she had experienced. A limitation with this procedure is that the examples given to the patients could have influenced the types of stressors they disclosed. However, this exemplification was done in the same manner for all patients and should therefore not have resulted in differences between the ED and EDrec groups.

#### Study sample

When interpreting the findings from this study, it is important to consider that the patients referred to the clinic were generally highly educated and that this may have influenced the types of stressors reported. It is plausible that individuals with lower socioeconomic status would report other stressors more frequently, such as financial worries. Moreover, since the patients in this study had been referred to a specialist clinic, it is possible that they represent more severe cases of ED compared with ED patients in primary care. Thus, future research needs to validate the results from the present study in a more heterogenous sample of ED patients. A final remark on the sample for this study is that it included fewer men than women. Due to the small number of participating men in this study, we did not perform any analyses regarding sex differences. Sex may, however, have an impact on experiences and diagnosis of ED, particularly given that women are more likely to be the primary carer for children and parents with long-term illnesses and disabilities, and so experience the compound impact of work-related and private-related stress [32, 33]. Future research should include comparative analyses of men and women with ED over time.

### Clinical implications

In the present study we found that adverse childhood experiences are similar among patients with long-term exhaustion and recovered patients. For clinical practice, this implies that the prognosis for individuals suffering from ED should not be determined based on their history of childhood adversities. Stress related to being a caregiver of a child with a psychiatric disorder or chronic illness was more frequently reported by the non-recovered group. This finding highlights the importance of identifying this subgroup of patients in clinical practice and to address their challenging situation during treatment. Our study also shows that this stressor may increase over time, and lead to a lack of recovery from ED. Hence, it is important to identify this stressor not only at baseline, but also throughout treatment. Another clinical implication of these results is that they underscore the importance of a well-functioning child and youth psychiatry and health care system. It is important to note, however, that ED is a clinical diagnosis and so treatment is on an individual basis. Unfortunately, the clinical treatment of individuals does little to change the structural issues that contribute to the development of this condition, particularly in parents of children with long-term illnesses and disabilities. Future research should explore the interplay between social and personal factors in the development of ED and consider how the provision of better structural support for carers and their children might prevent the onset and maintenance of this disorder. Our results also suggest that managerial responsibilities could be given special attention, since adding stress due to managerial responsibilities to a patient suffering from ED could delay recovery. Finally, this study indicates that it is the present stressors that are important for ongoing symptoms of ED. Neither adverse childhood experiences nor any of the stressors at baseline were associated with long-term ED. In contrast, stressors present at follow-up, such as caregiver stress (child) and managerial responsibilities, were more common in the ED group, and thus associated with persistent symptoms of ED. Hence, our study suggests a temporal relationship between the burden of stress exposure and the symptoms of exhaustion. This implies that the focus should be on the present situation and not on the past.

In this study we have explored some of many plausible factors that could be related to long-term exhaustion. Several other factors could also be related to long-term exhaustion and should be explored, one being personality traits. Thus, a recent study from our research group found that obsessive–compulsive personality disorder (OCPD), assessed by a self-reported screening questionnaire, was significantly more common among patients with ED who had not recovered 7–10 years after diagnosis compared to former ED patients that had recovered [34]. Taken together, it is plausible that, for some individuals, both personality traits and external stressors contribute to the long-term exhaustion to different degrees.

## Conclusions

The main conclusion of this study is that neither adverse childhood experiences nor any of the stressors at baseline are associated with long-term ED. A subgroup of patients who still fulfil the criteria for ED seven years after diagnosis report stress due to being a caregiver to a child with chronic disease or psychiatric disorder and this could plausibly contribute to long-lasting exhaustion for some of these patients. For clinical practice, our data suggest that the focus should be on reducing present stressors. Stress due to caring for a child with psychiatric disorders or chronic disease should be given special attention, as this stressor may be associated with long-term exhaustion.

## Data Availability

The datasets used and analysed during the current study are available from the corresponding author on reasonable request.

## Abbreviations

ED: Exhaustion Disorder
SD: Standard Deviation
CI: Confidence Intervals
